# Mid-clavicle fracture with dislocation of the ipsilateral acromioclavicular joint treated with Endobutton system: A case report and review of the literature

**DOI:** 10.1097/MD.0000000000027894

**Published:** 2021-11-24

**Authors:** Zhixiang Gao, Peng Cai, Kai Yao, Nengji Long, Lijuan Liu, Cong Xiao

**Affiliations:** Department of Orthopedics, The Third Hospital of Mianyang Sichuan Mental Health Center, No. 190 The East Jiannan Road, Mianyang, China.

**Keywords:** acromioclavicular joint, Endobutton, mid-clavicle

## Abstract

**Rationale:**

Acromioclavicular joint (ACJ) dislocation combined with ipsilateral midclavicular fracture is extremely unusual and is a high-energy injury to the shoulder. A review of the literature divides the treatment of clavicular fractures is divided into nonsurgical treatment, plates, and intramedullary nailing, while the options for ACJ dislocation are elastic fixation and rigid fixation. However, there is still a lack of consensus about the most appropriate way to treat this shoulder injury. This case report involves a mid-clavicle fracture with dislocation of the ipsilateral ACJ, which was classified as type V according to Rockwood classification.

**Patient concerns:**

A 65-year-old man came to the emergency department after a traffic accident in which he was driving a motorcycle that collided with another motorcycle and his right shoulder collided directly with the ground. Digital radiography (DR) and computed tomography (CT) scans of the right shoulder joint showed mid-clavicle fracture with dislocation of the ipsilateral ACJ, which was classified as type V according to Rockwood classification.

**Diagnoses:**

The diagnosis of mid-clavicle fracture with dislocation of the ipsilateral ACJ was confirmed by DR and CT.

**Interventions:**

The patient was treated with a clavicle plate combined with the double Endobutton technique.

**Outcomes:**

After a 6-month follow up, the patient had excellent results for shoulder range of motion and functional. The patient's Constant-Murley score is 92.

**Lessons:**

Mid-clavicular fracture with a high-energy injury is highly suspicious and requires perfect shoulder CT or bilateral shoulder stress position DR to confirm whether there is a combined ACJ dislocation.

## 1 Introduction

Clavicle fracture and acromioclavicular joint (ACJ) dislocation are common injuries in the shoulder; of these, clavicle fracture accounts for approximately 6% of systemic fractures and 44% of shoulder trauma, and ACJ dislocation accounts for approximately 3.2% of systemic joint dislocation and approximately 12% of shoulder trauma.^[1–6]^ However, ACJ dislocation combined with ipsilateral midclavicular fracture is extremely unusual and is a high-energy injury to the shoulder. If this injury occurs and the ACJ dislocation is not properly managed in time, it may cause shoulder joint dysfunction. A review of the literature highlighted 21 English-language articles reporting 26 cases as of August 2021 (Table 1). The surgical treatment of this shoulder injury has been described in the literature. The treatment of clavicular fractures is divided into nonsurgical treatment, plates, and intramedullary nailing, while the options for ACJ dislocation are elastic fixation (TightRope fixation system,^[7,8]^ dog bone button,^[9]^ reconstruction with tendon allograft,^[10]^ or preloaded suture fixation^[11]^) and rigid fixation (Kirschner wires,^[12–15]^ clavicular hook plate,^[16–20]^ and screw fixation^[21–23]^) or a combination of both.^[24]^ However, there is still a lack of consensus about the most appropriate way to treat this shoulder injury.

**Table 1:** Case report of a mid-clavicular fracture with ipsilateral ACJ dislocation.

			ACJ dislocation	Clavicle fracture (mid-shaft)			
Author, yr	n	Mechanism of injury	Type	Treatment	Types of fixation	Treatment	Follow-up
Sandesh Madi 2015^[9]^	1	Traffic accident	Type IV	The dog bone button	Elastic fixation	Locking plate	13 months; the patient's Constant-Murley score is 88. Returned to preinjury levels of sporting activities (cricket)
Spyridon A. 2011^[7]^	1	Traffic accident	Type V	TightRope fixation system	Elastic fixation	Locking plate	18 months; painless full ROM
Grossi 2013^[12]^	1	Fall from bicycle	Type VI	Fixed using 2 Steinmannwires	Rigid fixation	Nonoperative	12 months
Lancourt 1990^[13]^	1	Fall from horse	-	Fixed using 2 Steinmann wires	Rigid fixation	Nonoperative	3 years ;painless full ROM
Sharma N 2016^[14]^	1	Traffic accident	Type III	Kirschner wires	Rigid fixation	Locking plate	6 months; the patient's Constant-Murley score is 92
Beytemür 2013^[16]^	1	Traffic Steinmann wires accident	Type III	Clavicular hook plate	Rigid fixation	Locking plate	23 months; painless full ROM
Tidwell 2014^[21]^	1	Traffic accident	Type IV	Screw fixation	Rigid fixation	Locking plate	1 year; the patient reported intermittent soreness over the shoulder
Paryavi 2013^[17]^	1	Traffic accident	Type IV	Clavicular hook plate	Rigid fixation	Locking plate	8 months; DASH score:22
Rajeshkumar 2017^[8]^	1	Traffic accident	Type IV	Tight rope fixation system	Elastic fixation	Locking plate	5 months; DASH score: 11.7
Wurtz 1992^[22]^	1	Fall from bicycle	Type IV	Screw fixation	Rigid fixation	Nonoperative	3 years; painless full ROM
	1	Traffic accident	Type IV	Screw fixation	Rigid fixation	Nonoperative	2 years; painless full ROM
	1	Fall from horse	Type IV	Screw fixation	Rigid fixation	Nonoperative	3 years; painless full ROM
	1	Fall from horse	Type III	Nonoperative	-	Nonoperative	1 year; painless full ROM
Yeh 2009^[10]^	1	Fall from horse	Type IV	Reconstruction with tendon allograft	Elastic fixation	Locking plate	24 months; painless full ROM
Heinz 1995^[30]^	1	Fall from bicycle	Type II	Nonoperative	-	Nonoperative	24 months; returned to cycling, weight lifting, and competitive rowing
Wisniewski 2004^[15]^	1	Traffic accident	Type VI	Kirschner wires	Rigid fixation	Nonoperative	10 years; painless full ROM
Dong 2017^[18]^	1	Traffic accident	Type IV	Clavicular hook plate	Rigid fixation	Locking plate	1 year; painless full ROM
Solooki 2014^[23]^	1	Traffic accident	Type III	Screw fixation	Rigid fixation	Locking plate	1 year; painless full ROM
Davies 2014^[31]^	1	Fall from stairs	Type VI	Nonoperative		Locking plate	9 months; painless full ROM
Schots 2020^[24]^	1	Fall from height	Type IV	UltraPro composite mesh	Elastic fixation + Rigid fixation	Locking plate	6 months; regain full ROM but suffered from ongoing pain at the side of the AC joint
	1	Fall from bicycle	Type III	UltraPro composite mesh	Elastic fixation + Rigid fixation	Locking plate	7 months; painless full ROM
Juhn 2002^[32]^	1	Struck the boards with his shoulder	Type VI	Nonoperative	-	Nonoperative	10 months; returned to play ice hockey free of shoulder or clavicle symptoms
Wijdicks 2013^[19]^	1	Traffic accident	Type III	Clavicular hook plate	Rigid fixation	Locking plate	13 months; DASH score: 3.33
	1	Traffic accident	Type IV	Clavicular hook plate	Rigid fixation	Locking plate	6 months; painless full ROM
Woolf 2013^[20]^	1	Traffic accident	Type IV	Clavicular hook plate	Elastic fixation	Locking plate	3 years; painless full ROM
López Palacios 2021^[11]^	1	Traffic accident	Type IV	Preloaded suture fixed	Elastic fixation	Locking plate	30 months; the patient's Constant-Murley score is 97

DASH = disabilities of the arm, shoulder, and hand, ROM = range of motion.

We report a case of mid-clavicle fracture combined with ipsilateral ACJ dislocation, which was treated with a clavicle plate combined with the double Endobutton technique. We have also reviewed the cases reported in the international literature and analyzed them in terms of the cause of injury, type of dislocation, and treatment options.

This anonymous case report was published with the consent of the patient and his family. Ethical approval was attained by the third hospital of mianyang ethical approval board.

## 2 Case report

**Figure 1:**
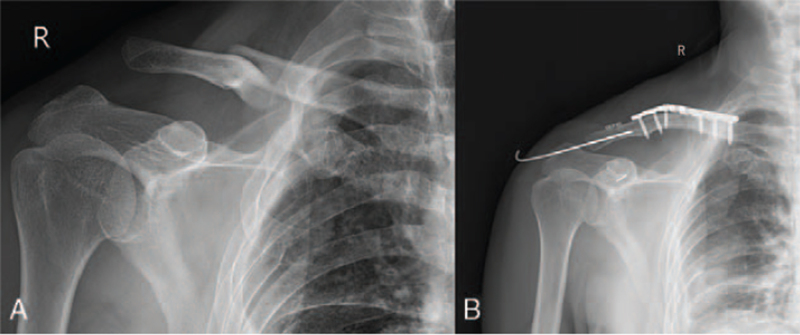
A. Anteroposterior digital radiography (DR) of the right shoulder shows mid-third clavicle fracture with ipsilateral type V ACJ dislocation (according to Rockwood classification). B. The postoperative radiograph showing anatomical reduction in the ACJ. ACJ = acromioclavicular joint.

A 65-year-old man with swelling and tenderness in his right shoulder and chest came to the emergency department after a traffic accident in which he was driving a motorcycle that collided with another motorcycle and his right shoulder collided directly with the ground. Although there were deformities and tenderness in the middle and lateral ends of the clavicle and an inability to use the right shoulder, there was no neurovascular injury. Digital radiography (DR) and computed tomography (CT) scans of the right shoulder joint showed mid-clavicle fracture with dislocation of the ipsilateral ACJ, which was classified as type V according to Rockwood classification (Fig. 1A). Further examination also revealed mild right hemopneumothorax and multiple rib fractures on the right side that were treated conservatively.

**Figure 2:**
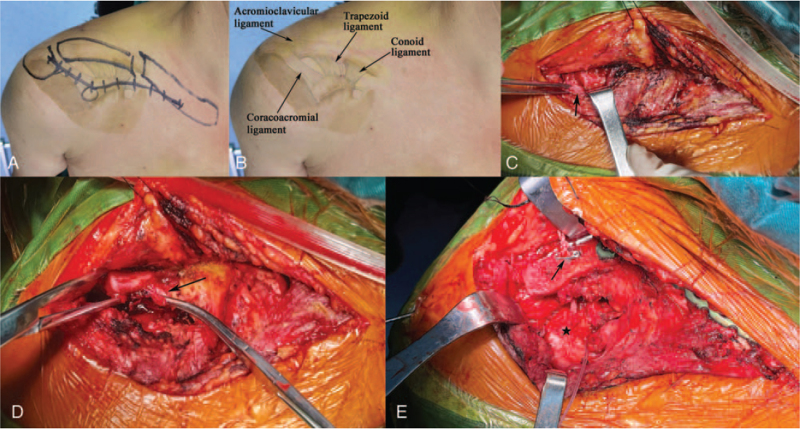
A and B. Design and landmarks of the surgical incision. C. Exposure of the ACJ; black arrows represent the intra-articular fibrocartilage disc. D. The black arrow indicates the ruptured coracoclavicular ligaments. E. After the ACJ has been reset; the black arrow represents the EndoButton, and the black pentagram represents the coracoid process. ACJ = acromioclavicular joint.

The surgery was performed under general anesthesia induction, whereas the patient was positioned as if seated in a beach chair. The shapes of the acromioclavicular joint, sternoclavicular joint, coracoid process, and clavicle were determined, and an “S”-shaped surgical incision was made through the tip of the coracoid process (Fig. 2A). All soft tissues were removed from the clavicle fracture. The ruptured acromioclavicular ligament and intra-articular fibrocartilage disc were cleared (Fig. 2C). The anterior deltoid was split longitudinally, and the distal clavicle was pulled posteriorly upward to clear the ruptured coracoclavicular ligaments (trapezoid ligament and conoid ligament) (Fig. 2D). After the clavicle fracture was reset, a locking plate with 8 holes was placed over the clavicle for fixation. A 2.0 mm drill guide was inserted into the clavicle at a distance of 2.5 cm from the ACJ,^[25]^ and after confirmation that the tip of the guide wire was located in the coronoid process, drilling to the base of the coronoid process was continued. Then, through the guidewire, the coracoid process and clavicle tunnels were created with a 4.0 mm cannulated drill. The 3.5 mm Endobutton (3.8 × 1.2 mm, Johnson & Johnson, Switzerland) was selected. The Endobutton was retracted along the bone tunnel from below the coronoid process to above the clavicle, and pressure was applied to reset the ACJ. Finally, the 2 ends were tied together with a surgeon's knot and 3 square knots while maintaining the ACJ reset. The final examination revealed ACJ anterior–posterior instability; therefore, the ACJ was fixed by introducing a 2.0 mm smooth Kirschner wire from the acromion to the lateral clavicle (Fig. 2E). The acromioclavicular ligament, coracoclavicular ligaments, and acromioclavicular capsule were repaired using absorbable sutures. The patient's total intraoperative blood loss was 80 mL and postoperative hospital stay was 6 days. Figure 1B shows a postoperative radiograph showing anatomical reduction in the ACJ. At the 6-month postoperative follow-up, patients were scored 92 on the Constant-Murley by telephone.

## 3 Discussion

**Figure 3:**
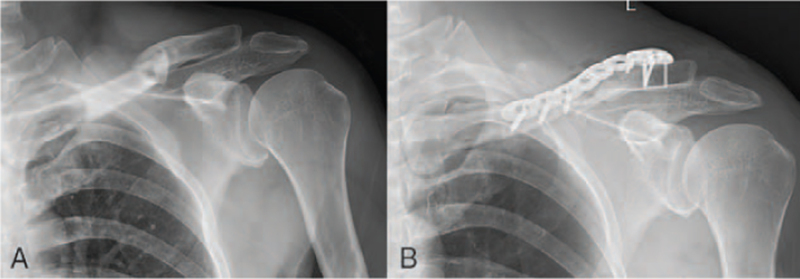
A. Preoperative anteroposterior shoulder joint DR shows a mid-third clavicle fracture without ipsilateral ACJ dislocation. B. Evidence of ACJ subluxation at the 1-month postoperative follow-up of clavicle fracture. ACJ = acromioclavicular joint; DR = digital radiography.

Fracture of the mid-third of the clavicle with ipsilateral ACJ dislocation is a rare high-energy floating clavicle injury. Its incidence does not appear to be very high, and it has been reported only in case reports. However, Ottomeyer et al^[26]^ investigated 183 surgically treated and 200 nonsurgically treated patients with mid-clavicle fractures and found a total of 26 cases of combined ipsilateral ACJ dislocation with an incidence of 6.8%, including 13 cases (7.1%) in the surgical group and 23 cases (6.5%) in the nonoperative group. More interestingly, only 2 cases of combined ACJ dislocation in the operative group were recognized preoperatively, while the others were identified during the postoperative follow-up. Exactly the same thing occurred in our postoperative follow-up of the clavicle fracture. Preoperative DR showed a clavicle fracture (Fig. 3A), but there was evidence of ACJ dislocation (type II) at the 1-month postoperative follow-up (Fig. 3B). Hence, to diagnose this class of injury accurately at an earlier stage and avoid a misdiagnosis, the author suggested that the following points need to be noted: for all mid-clavicular fractures, a physical examination of the ACJ and coracoid process is required; if there is pressure pain, ACJ injury is highly suspected, and the “Piano Key sign” test is not recommended. Mid-clavicular fracture with a high-energy injury is highly suspicious and requires perfect shoulder CT or bilateral shoulder stress position DR. Notably, Rockwood IV ACJ dislocation is shown as an enlarged ACJ gap on DR, which is easily missed, and CT of the shoulder joint is recommended for a definitive diagnosis. The stability of the ACJ should be checked after the intraoperative fixation of the mid-clavicular fracture is finished, and if necessary, fluoroscopy of the ACJ under traction with the C-arm should be performed. If a dislocation is found, it can be dealt with time.

We retrieved related literature via PubMed, EMBASE, Google, Web of Science by using keywords “Mid-clavicle,” and “Acromioclavicular joint.” Cases in the literature reporting mid-clavicle fractures with ipsilateral ACJ dislocation were included in the study. We analyzed the causes of injury by reviewing 26 patients in 21 articles in the literature: most of the clavicle fractures with ipsilateral ACJ dislocation were high-energy injuries, including 15 cases of traffic accident, 4 cases of fall from a bicycle, 4 cases of fall from a horse, 1 case of fall from a height, 1 case of fall from the stairs, and 1 case in which the patient struck boards with his shoulder. Therefore, for the high-energy injury of clavicle fractures mentioned above, traffic accidents should alert physicians to ACJ dislocation. However, the mechanism of occurrence has not been explained in detail, and it is unclear which occurs first, midclavicular fracture, ACJ dislocation, or both simultaneously. Marjoram and Chakrabarti^[27]^ believe that the high-energy impact first hits the ACJ, causing dislocation of the ACJ, which then continues to spread inward along the clavicle axis, eventually leading to clavicle fracture due to the mechanical weakness of the mid-third of the clavicle. Maruyama et al^[28]^ and Okano et al^[29]^ suggested that the “first rib pivot theory” could explain the occurrence of a “floating clavicle,” such as a medial or mid-third clavicle fracture with ipsilateral ACJ dislocation. When the shoulder was struck with force, the scapula and clavicle moved together posteriorly and inferiorly relative to the trunk, which could result in ACJ dislocation. The clavicle stops moving downward due to contact with the first rib and is subjected to downward external force and tension from the deltoid muscle, and the clavicle fractures with the first rib as the pivot/stress point.

Previous reports of mid-third clavicle fractures with ipsilateral ACJ dislocation have described various treatment options. At present, there is a consensus on the treatment options for mid-third clavicle fractures. Therefore, such injuries can be treated as a single ACJ dislocation after clavicle fracture fixation. Treatment options for ACJ dislocation are guided by the Rockwood classification: for Type I and Type II, opt for conservative treatment; treatment of Type III dislocations is controversial; and surgical management is recommended for Type IV, Type V, and Type VI. The 26 patients reported in the literature included 1 case of Type II,^[9]^ which was treated conservatively; 6 cases of type III,^[14,16,19,23,30]^ 13 cases of type IV,^[8–11,17,18,20–22,24]^ 1 case of type V,^[7]^ and 4 cases of type VI^[12,15,31,32]^ were treated with surgery. In these patients with rigid fixation, the fixative was removed after approximately 8 weeks to 8 months. Woolf et al^[20]^ reported a case of mid-clavicle fracture with dislocation of the ipsilateral ACJ managed with a clavicle hook plate, in which the patient developed persistent ACJ pain postoperatively, the clavicle hook plate was removed 24 weeks after surgery. The pain improved 2 weeks later, and there was no shoulder movement disorder 3 years later. Tidwell et al^[21]^ reported that 1 year after the initial surgery, the patient complained of intermittent pain in the lateral shoulder that was not limited to movement, and the pain symptoms resolved after removal of the coracoclavicular screw; however, the subluxation of the ACJ had not changed significantly at 3 months of follow-up.

The literature reports good clinical results of the double Endobutton technique in the treatment of single ACJ dislocations.^[33,34]^ The Endobutton was retracted along the bone tunnel from below the coronoid process to above the clavicle, and pressure was applied to reset the ACJ. Finally, the 2 ends were tied together with a surgeon's knot and 3 square knots while maintaining the ACJ reset. This technique does not interfere with the rotator cuff, does not affect the function of the shoulder joint, and does not have many of the problems associated with tendon autograft or allograft reconstruction methods. Therefore, this method is now widely used.

To the best of our knowledge, this is the first case in which a double Endobutton was used to treat a mid-clavicle fracture with dislocation of the ipsilateral ACJ. Additionally, there was no restriction on the location of the clavicle fracture site with the Endobutton. If the distal clavicle plate occupies the position of the Endobutton, it is still possible to fix the Endobutton to the clavicle plate hole by reserving a hole in the clavicle plate. In our patient, the Kirschner wires was removed 6 weeks after surgery. The patient had excellent short-term results for shoulder range of motion and functional.

## Acknowledgments

The authors thank AJE for its linguistic assistance during the preparation of this manuscript.

## Author contributions

**Investigation:** Nengji Long, Lijuan Liu.

**Supervision:** Cong Xiao.

**Writing – original draft:** Zhixiang Gao.

**Writing – review & editing:** Peng Cai, Kai Yao, Cong Xiao.

## References

[1] Ellis HB Li Y Bae DS. Descriptive epidemiology of adolescent clavicle fractures: results from the FACTS (Function after Adolescent Clavicle Trauma and Surgery) prospective, multicenter cohort study. Orthop J Sports Med 2020;8:2325967120921344. 32528990 10.1177/2325967120921344 PMC7263158

[2] McCarthy MM Bihl JH Frank RM Salem HS McCarty EC Comstock RD. Epidemiology of clavicle fractures among US High School athletes, 2008-2009 through 2016-2017. Orthop J Sports Med 2019;7:2325967119861812. 31384623 10.1177/2325967119861812 PMC6661795

[3] Kihlström C Möller M Lönn K Wolf O. Clavicle fractures: epidemiology, classification and treatment of 2 422 fractures in the Swedish Fracture Register; an observational study. BMC Musculoskelet Disord 2017;18:82. 28202071 10.1186/s12891-017-1444-1 PMC5312264

[4] Herteleer M Winckelmans T Hoekstra H Nijs S. Epidemiology of clavicle fractures in a level 1 trauma center in Belgium. Eur J Trauma Emerg Surg 2018;44:717–26. 29027569 10.1007/s00068-017-0858-7

[5] Oberle L Pierpoint L Spittler J Khodaee M. Epidemiology of clavicle fractures sustained at a Colorado Ski resort. Orthop J Sports Med 2021;9:23259671211006722. 34026919 10.1177/23259671211006722 PMC8120545

[6] Chillemi C Franceschini V Dei Giudici L. Epidemiology of isolated acromioclavicular joint dislocation. Emerg Med Int 2013;2013:171609. 23431452 10.1155/2013/171609 PMC3568861

[7] Psarakis SA Savvidou OD Voyaki SM Beltsios M Kouvaras JN. A rare injury of ipsilateral mid-third clavicle fracture with acromioclavicular joint dislocation. Hand (N Y) 2011;6:228–32. 22654711 10.1007/s11552-011-9323-y PMC3092883

[8] Kakwani RG Tourret LJ. Fracture clavicle with acromioclavicular dislocation: a complex injury. Shoulder Elbow 2017;3:31–3.

[9] Madi S Pandey V Khanna V. A dual injury of the shoulder: acromioclavicular joint dislocation (type IV) coupled with ipsilateral mid-shaft clavicle fracture. BMJ Case Rep 2015;2015:bcr2015213254. 10.1136/bcr-2015-213254 PMC4680282 26598529

[10] Yeh PC Miller SR Cunningham JG Sethi PM. Midshaft clavicle fracture and acromioclavicular dislocation: a case report of a rare injury. J Shoulder Elbow Surg 2009;18:e1–4. 10.1016/j.jse.2008.09.011 19046642

[11] López Palacios C Sanchez-Munoz E Pipa Muñiz I Rodríguez García N Maestro Fernández A. Simultaneous clavicle fracture and acromioclavicular joint dislocation: novel surgical technique: a case report. JBJS Case Connect 2021;11:02. 10.2106/JBJS.CC.20.00775 34161305

[12] Grossi EA Macedo RA. Acromioclavicular dislocation type VI associated with diaphyseal fracture of the clavicle ∗. Rev Bras Ortop 2013;48:108–10. 31304120 10.1016/j.rboe.2011.12.002 PMC6565910

[13] Lancourt JE. Acromioclavicular dislocation with adjacent clavicular fracture in a horseback rider. A case report. Am J Sports Med 1990;18:321–2. 2372085 10.1177/036354659001800317

[14] Sharma N Mandloi A Agrawal A Singh S. Acromioclavicular joint dislocation with ipsilateral mid third clavicle, mid shaft humerus and coracoid process fracture - a case report. J Orthop Case Rep 2016;6:24–7. 10.13107/jocr.2250-0685.414 PMC5040563 27703932

[15] Wisniewski T. Posterior acromioclavicular dislocation with clavicular fracture and trapezius entrapment. Eur J Trauma 2004;30:120–3.

[16] Beytemür O Adanir O Dinel YM Baran MA Güle MA. Clavicle diaphyseal fracture, ipsilateral type 3 acromioclavicular joint dislocation stabilized with double plate. Int J Shoulder Surg 2013;7:153–4. 24403764 10.4103/0973-6042.123536 PMC3883191

[17] Paryavi E Christian MW Pensy RA Eglseder WA. Floating clavicular injury: treatment of combined midshaft fracture and acromioclavicular separation with a dual plating technique. Curr Orthop Pract 2013;24:349–52.

[18] Dong D Yu M Gu G. Simultaneous bilateral midshaft clavicle fractures with unilateral dislocation of the acromioclavicular joint: a case report. Medicine (Abingdon) 2017;96:e6975. 10.1097/MD.0000000000006975 PMC5457878 28538398

[19] Wijdicks CA Anavian J Ly TV Spiridonov SI Craig MR Cole PA. Surgical management of a midshaft clavicle fracture with ipsilateral acromioclavicular dislocation: a report on 2 cases and review of the literature. Injury Extra 2013;44:09–12.

[20] Woolf SK Valentine BJ Ba Rfield WR Hartsock LA. Middle-third clavicle fracture with associated type IV acromioclavicular separation: case report and literature review. J Surg Orthop Adv 2013;22:183–6. 23628577 10.3113/jsoa.2013.0183

[21] Tidwell JE Kennedy PM Mcdonough EB. Concurrent treatment of a middle-third clavicle fracture and type IV acromioclavicular dislocation. Am J Orthop 2014;43:E275–8. 25379757

[22] Wurtz LD Lyons FA Rockwood CA. Fracture of the middle third of the clavicle and dislocation of the acromioclavicular joint. A report of four cases. J Bone Joint Surg Am 1992;74:133–7. 1734003

[23] Solooki S Azad A. Simultaneous middle third clavicle fracture and type 3 acromioclavicular joint dislocation; a case report. Arch Bone Joint Surg 2014;2:69–71. 25207318 PMC4151440

[24] Schots JP van Laarhoven SN Hustinx PA Pijnenburg AM Meesters B de Loos ER. Surgical treatment of acromioclavicular dislocation associated with midshaft fracture of the ipsilateral clavicle. Acta Orthop Belg 2020;86:532–8. 33581039

[25] Struhl S. Double Endobutton technique for repair of complete acromioclavicular joint dislocations. Tech Shoulder Elbow Surg 2007;8:175–9.

[26] Ottomeyer C Taylor BC Isaacson M Martinez L Ebaugh P French BG. Midshaft clavicle fractures with associated ipsilateral acromioclavicular joint dislocations: incidence and risk factors. Injury 2017;48:469–73. 28062098 10.1016/j.injury.2016.12.021

[27] Marjoram TP Chakrabarti A. Segmental clavicle fracture and acromio-clavicular joint disruption: an unusual case report. Shoulder Elbow 2015;7:187–9. 27582977 10.1177/1758573214564496 PMC4935151

[28] Maruyama K Sugawara R Sano S. "Similar case of panclavicular dislocation”. Katakansetsu 1984;8:147–50.

[29] Okano I Sawada T Inagaki K. Bipolar dislocation of the clavicle: a report of two cases with different injury patterns and a literature review. Case Rep Orthop 2017;2017:2935308. 29527368 10.1155/2017/2935308 PMC5763060

[30] Heinz WM Misamore GW. Mid-shaft fracture of the clavicle with grade III acromioclavicular separation. J Shoulder Elbow Surg 1995;4:141–2. 7600166 10.1016/s1058-2746(05)80069-2

[31] Davies EJ Fagg JA Stanley D. Subacromial, supracoracoid dislocation of the acromioclavicular joint with ipsilateral clavicle fracture: a case report with review of the literature and classification. J R Soc Med Cardiovasc Dis 2014;5:2054270414527281. 10.1177/2054270414527281 PMC4100230 25057405

[32] Juhn MS Simonian PT. Type VI acromioclavicular separation with middle-third clavicle fracture in an ice hockey player. Clin J Sport Med 2002;12:315–7. 12394206 10.1097/00042752-200209000-00011

[33] Spoliti M De Cupis M Via AG Oliva F. All arthroscopic stabilization of acute acromioclavicular joint dislocation with fiberwire and endobutton system. Muscles Ligaments Tendons J 2014;4:398–403. 25767774 PMC4327346

[34] Struhl S Wolfson TS. Continuous loop double Endobutton reconstruction for acromioclavicular joint dislocation. Am J Sports Med 2015;43:2437–44. 26260466 10.1177/0363546515596409

